# Disrupted white matter integrity and network connectivity are related to poor motor performance

**DOI:** 10.1038/s41598-020-75617-1

**Published:** 2020-10-27

**Authors:** Feifei Zhai, Jie Liu, Ning Su, Fei Han, Lixin Zhou, Jun Ni, Ming Yao, Shuyang Zhang, Zhengyu Jin, Liying Cui, Feng Tian, Yicheng Zhu

**Affiliations:** 1 Department of Neurology, Peking Union Medical College Hospital, Peking Union Medical College and Chinese Academy of Medical Science, No.1 Shuaifuyuan, Wangfujing, Beijing, 10073 China; 2 State Key Laboratory of Computer Science and Beijing Key Lab of Human-Computer Interaction, Institute of Software, Chinese Academy of Sciences, Beijing, China; 3 School of Artificial Intelligence, University of Chinese Academy of Sciences, Beijing, China; 4 Department of Cardiology, Peking Union Medical College Hospital, Peking Union Medical College and Chinese Academy of Medical Science, Beijing, China; 5 Department of Radiology, Peking Union Medical College Hospital, Peking Union Medical College and Chinese Academy of Medical Science, Beijing, China

**Keywords:** Motor control, Neural ageing

## Abstract

Motor impairment is common in the elderly population. Disrupted white matter tracts and the resultant loss of connectivity between cortical regions play an essential role in motor control. Using diffusion tensor imaging (DTI), we investigated the effect of white matter microstructure on upper-extremity and lower-extremity motor function in a community-based sample. A total of 766 participants (57.3 ± 9.2 years) completed the assessment of motor performance, including 3-m walking speed, 5-repeat chair-stand time, 10-repeat hand pronation-supination time, and 10-repeat finger-tapping time. Fractional anisotropy (FA), mean diffusivity (MD), and structural network connectivity parameters were calculated based on DTI. Lower FA and higher MD were associated with poor performance in walking, chair-stand, hand pronation-supination, and finger-tapping tests, independent of the presence of lacunes, white matter hyperintensities volume, and brain atrophy. Reduced network density, network strength, and global efficiency related to slower hand pronation-supination and finger-tapping, but not related to walking speed and chair-stand time. Disrupted white matter integrity and reduced cerebral network connectivity were associated with poor motor performance. Diffusion-based methods provide a more in-depth insight into the neural basis of motor dysfunction.

## Introduction

Motor deterioration is common in the elderly population and poses a public health threat in light of the rapidly increasing aging population^1,2^. Impaired function of lower limbs was related to poor gait, uncontrolled balance, and increased risk of falls^3^. Much of previous studies have investigated the neural correlates of lower extremity functions and revealed that lacunar infarcts^4,5^, white matter hyperintensities^4–12^, disrupted white matter integrity^13–18^, and brain atrophy^7,11,19,20^ were associated with reduced walking speed or impaired balance control. Compared with the lower extremity, manual dexterity was more important for fine motor skills in daily activities, such as feeding, dressing, and grooming, especially in the elderly^21^. Although sparse, exiting findings have also linked poor manual dexterity and worse finger task performance to greater white matter hyperintensities (WMH)^22,23^ volume and disrupted white matter tract integrity^24,25^. Few studies have investigated how upper extremity and lower extremity functions relate to brain structural lesions in the same sample^26^. Examing these associations in the same sample allows for a comparison of different motor tasks and their neural correlates.

Motor control requires receiving and processing various input information, including visual, proprioceptive, vestibular, and sensorimotor information. To properly integrate these inputs and accurately plan and control the movement, many cortical and subcortical regions interact delicately with each other through white matter networks^2,27–29^. Damage to white matter tracts possibly impedes communication between these networks, which might result in motor impairment^7,8,18,30^. Growing evidence shows that changes in macrostructural white matter changes on conventional magnetic resonance imaging (MRI), such as WMH, white matter atrophy, and lacunes, are related to declined motor performance^4,5,10–12^. However, such conventional markers only represent the tip of the iceberg about changes in white matter^31^. Focusing on microstructural changes using the white matter integrity measured by diffusion-weighted imaging, may provide a more in-depth insight of alteration in the white matter. Diffusion tensor imaging (DTI) allows the mapping of the diffusion of water molecules along the white matter fiber in the brain and enables quantification of white matter microstructural organization in vivo, including normal-appearing white matter^32^. Reduced fractional anisotropy (FA) and increased mean diffusivity (MD) are generally regarded as disrupted fiber tracts and demyelination, which are more sensitive in the early detection of changes in white matter microstructure^33^. A variety of microstructural white matter changes have been identified in normal aging both in cross-sectional^34–36^ and longitudinal^37,38^ studies. Our previous study found that WMH and brain atrophy were associated with poor motor performance in the general population^26^. Further quantification of microstructural organization and connectivity of white matter in the brain will provide new insight into the biological basis of motor dysfunction.

In this study, we used DTI and deterministic tractography to characterize white matter integrity and network topology in a population-based sample. We aimed to investigate the effect of white matter integrity and network connectivity on four objective measures of both upper and lower extremities motor functions (walking speed, chair-stand time, hand pronation-supination, and finger-tapping), beyond conventional MRI markers.

## Methods

### Population

This study was a cross-sectional analysis of an ongoing prospective community-based Shunyi cohort study that included participants aged 35 years and older^39^. From June 2013 to April 2016, 1586 individuals completed standard baseline assessments. Among these persons, 909 participants had undergone both brain MRI examination and motor function evaluation. In the present study, we excluded 58 participants with prior stroke and 14 with muscle strength levels lower than grade 3 or with involuntary movements (tremor or chorea). MRI quality was visually assessed, and participants with poor structural MRI quality (technical artifacts, n = 3; susceptibility artifact, n = 5; motion artifacts or radiofrequency noise, n = 54), atypical structures (large subarachnoid cyst, n = 3; big ventricles, n = 2), and inadequate DTI scans (n = 4) were further excluded, leaving 766 participants for this analysis. The study was approved by the Medical Review Ethics Committee of Peking Union Medical College Hospital (reference number: B-160) and all methods were performed in accordance with relevant guidelines and regulations. All participants signed an informed consent form on their own.

### Measurement of motor performance

Motor performance was quantified using standard motor scales, which have been described elsewhere^26,40^. The lower-extremity function was evaluated via 3-m walking speed and 5-repeat chair-stand time. The upper-extremity function was evaluated by 10-repeat hand pronation-supination time and 10-repeat finger-tapping time. For assessment of walking speed, participants were asked to walk at a usual pace over a 3-m distance twice, and the average speed was used in the analysis. In the chair-stand test, a subset of the Short Physical Performance Battery (SPPB)^41^, participants were asked to stand up and sit down five times as quickly as possible while keeping their arms folded across their chests. In the hand pronation-supination test, a subset of the Scale for the Assessment and Rating of Ataxia (SARA)^42^, participants were asked to perform ten cycles pronation and supination with each hand on their thigh as fast as possible. In the finger-tapping test, a subset from the Unified Parkinson’s Disease Rating Scale (UPDRS)^43^, participants were asked to tap the index finger on the thumb tip ten times as quickly and powerfully as possible. Because there were no significant differences in times between the left and right hand for either pronation-supination or finger-tapping tests, the scores for both hands were averaged in each task.

### MRI acquisition

MRI acquisition was performed using a single 3-T Siemens Skyra scanner (Siemens; Erlangen, Germany) at Shunyi Hospital. Three-dimensional T1-weighted images were acquired using a magnetization-prepared rapid gradient-echo (MPRAGE) sequence with the following parameters: 144 sagittal slices, voxel size = 1 × 1  × 1.3  mm^3^, repetition time (TR) = 2530 ms, echo time (TE) = 3.43 ms, inversion time = 1100 ms, field of view (FOV) = 256 × 256  mm^2^, flip angle = 8°. Fluid-attenuated inversion recovery (FLAIR) images were acquired with the following parameters: 80 axial slices, slice thickness = 5 mm, gap = 1 mm, TR = 8500 ms, TE = 81 ms, FOV = 230 × 230 mm^2^, flip angle = 150°. Diffusion-weighted images were acquired using a single-shot spin echo-planar imaging sequence covering the whole brain with the following parameters: 62 axial slices, slice thickness = 2.2 mm without gap; TR = 8000 ms, TE = 89 ms, flip angle = 90°, 30 diffusion-weighted directions with b = 1000 s/mm^2^ and one non-diffusion weighted image with b = 0 s/mm^2^, voxel size = 2.2 × 2.2 × 2.2 mm^3^, FOV = 280 × 280 mm^2^, average = 2. We did not acquire reversed phase encoding image in this study, and we used bipolar pulse sequence to reduce eddy current distortions.

### Diffusion tensor imaging processing and network reconstruction

Diffusion tensor imaging data preprocessing were performed using PANDA, which is a pipeline toolbox for diffusion MRI analysis^44^. Briefly, the preprocessing procedure included skull-stripping, eddy-current, and head-motion correction. Fractional anisotropy (FA) and mean diffusivity (MD) were created by fitting a diffusion tensor model. Then, voxelwise analysis of FA and MD was performed using tract-based spatial statistics (TBSS) in PANDA pipeline^44^. First, individual FA images were non-linearly registered to the FA standard template in Montreal Neurological Institute space. Next, the mean of all aligned FA images was calculated and skeletonized to create a mean FA skeleton. A threshold of 0.2 in FA was used to limit the analysis to major white matter tracts. Then, each participant’s highest FA value near the skeleton was projected onto the mean skeleton by calling *tbss_skeleton* command of FSL (https://fsl.fmrib.ox.ac.uk/fsl/fslwiki)^45^. Finally, voxelwise statistical analysis on the skeleton was performed.

We also performed whole-brain white matter tractography using deterministic fiber tracking via the Fiber Assignment by Continuous Tracking algorithm^46^. Streamlines were terminated when FA < 0.2 or turning angle > 60°. Then, we used the automated anatomical labeling (AAL) template^47^ to parcellate the cerebral cortex into 90 cortical and subcortical regions (45 for each hemisphere). Two regions were considered connected if the endpoints of the reconstructed fiber buddle lay within both regions^48^. For each participant, a weighted edge was constructed via multiplying the number of reconstructed fiber by the mean FA along the fiber bundle connecting the two regions^49,50^. The connection strength was further normalized by the average volume of each pair of regions to correct for different sizes of the AAL regions and different brain sizes^50,51^. This resulted in an undirected weighted 90 × 90 connectivity matrix for each participant. The average connectivity matrix was shown in Supplementary Fig. 1.

The topological properties of the white matter network were computed using brain connectivity toolbox^52^ (https://sites.google.com/site/bctnet/), based on graph theory^52^. Network density is defined as the total number of observed edges in a network divided by the possible number of edges. Total network strength is calculated as the sum of the weighted edges of a network. Global efficiency is defined as the inverse of the shortest path lengths, reflecting how efficiently information is exchanged over the network. To explore the location of network disruption, nodal efficiency was also computed. The nodal efficiency for a given node was defined as the inverse of the shortest path length between that node and all other nodes in the network, quantifying the importance of the nodes for communication within the network.

### Macrostructural neuroimaging markers

Lacunes were defined as focal fluid-filled cavities of 3 to 15 mm in diameter situated in the basal ganglia or subcortical white matter, according to the Standards for Reporting Vascular Changes on Neuroimaging^53^. The gray matter (GM), white matter (WM), and cerebrospinal fluid were automatically segmented on structure T1-weighted images using Statistical Parametric Mapping 12 (https://www.fil.ion.ucl.ac.uk/spm/) and CAT12 toolbox (https://www.neuro.uni-jena.de/vbm/). Total intracranial volume (TIV) was computed as the sum of the volumes of GM, WM, and cerebrospinal fluid. Brain parenchymal fraction (BPF), as a surrogate index of brain atrophy, was the ratio of brain tissue volume to TIV. WMH were automatically segmented by the lesion growth algorithm, as implemented in the lesion segmentation tool (LST) toolbox (https://www.statistical-modelling.de/lst.html) for Statistical Parametric Mapping.

### Other measurements

Cognitive status was evaluated by neurologists using the Mini-Mental State Examination (MMSE), which is a test with a total score ranging from 0 to 30. Hypertension was defined as self-reported hypertension, blood pressure ≥ 140/90 mmHg, or use of anti-hypertensive medication. Diabetes mellitus was defined as fasting serum glucose ≥ 7.0 mmol/L, self-reported diabetes mellitus, or use of oral antidiabetic drugs or insulin. Hyperlipidemia was defined as self-reported hyperlipidemia, total cholesterol > 5.2 mmol/L, low-density lipoprotein-cholesterol > 3.36 mmol/L, or use of lipid-lowering medication. Smoking status was classified into current smoking (at least within the past month) and noncurrent smoking.

### Statistical analysis

The first goal of the study was to evaluate whether topographical changes in white matter integrity were associated with motor performance. To achieve this goal, we performed TBSS to assess the associations of skeletal DTI parameters (FA and MD) with each motor parameter (walking speed, chair-stand time, pronation-supination time, and finger-tapping time) using general linear models, respectively. All statistical analyses included age, sex, height (only in gait velocity and chair-stand models), MMSE, the presence of lacunes, WMH volume (log-transformed), and BPF. All covariates were demeaned before entering into the models. TBSS was performed using a permutation-based statistical interference tool for nonparametric analyses as part of the FSL toolbox (https://fsl.fmrib.ox.ac.uk/fsl/fslwiki/Randomise). The number of permutation tests was set at 5000. Significant associations were determined using a threshold-free cluster enhancement with a p-value < 0.05 to correct for multiple testing^54,55^.

The second goal of the study was to investigate whether network connectivity was associated with motor performance. To achieve this goal, we used multiple linear regressions with each motor performance as the dependent variable and each global network parameter (network density, total network strength, global efficiency) as the independent variable respectively. Only one network connectivity parameter was included in the model at one time. We constructed 2 models. Model 1 adjusted for age, sex, height (only in gait velocity and chair-stand models), and MMSE. Model 2 additionally adjusted for the presence of lacunes, WMH volume, and brain parenchymal fraction. Collinearity was evaluated by the variance inflation factors (VIF). The VIF of all variables in the full models were < 5. Statistical significance was defined as two-sided p < 0.05. In post hoc analysis, we calculated partial correlation coefficients between 90 nodal efficiency and each motor parameter to evaluate the spatial distribution of nodes (brain regions) that were associated with poor motor performance. To account for multiple testing, the false discovery rate (FDR) adjustment was conducted at each motor performance level (i.e., walking speed, chair-stand time, pronation-supination time, and finger-tapping time) using Benjamini–Hochberg procedure with a 0.05 level (q value) as the critical value. Statistical analysis was conducted with SAS 9.4 (SAS Institute, Cary, NC).

To validate the robustness of our results, we examined the influence of different data analysis strategies as follows. First, from the perspective of neuroanatomy, a given hemisphere primarily controls the strength and movement of the contralateral limbs. Therefore, we conducted analyses on left- and right-hand pronation-supination and finger-tapping respectively instead of the averaged score. Second, cardiovascular risk factors are important causes of brain pathology; therefore, we additionally adjusted for cardiovascular risk factors, including hypertension, diabetes mellitus, hyperlipidemia, and current smoking.

## Results

The study included 766 participants. The mean age of study participants was 57.3 years (SD 9.2, range 35–82), and 267 (35%) were male. Table 1 shows demographic, neuroimaging, and motor performance characteristics. Compared with the rest of the cohort, individuals included in this study were younger (55.8 y vs. 57.3 y), comprised of fewer males (35% vs. 47%), and had better cognition (MMSE 26.3 vs. 25.4). Correlation analysis showed that advanced age was related to poor motor performance. White matter integrity and network connectivity measures were significantly correlated with WMH volume, the presence of lacunes, and brain parenchymal fraction. (Supplementary Table 1).

**Table 1:** Characteristics of the study population.

Variables	
**Demographics**	
Age, years	57.3 (9.2)
Male	267 (35%)
Height, cm	158.9 (7.8)
MMSE	26.3 (3.5)
Hypertension	380 (50%)
Diabetes mellitus	122 (16%)
Hyperlipidemia	369 (48%)
Current smoking	166 (22%)
**Motor performance**	
Walking speed, m/s	0.9 (0.2)
5-repeat chair-stand time, s	8.9 (2.1)
10-repeat pronation-supination time, s	7.5 (1.6)
10-repeat finger-tapping time, s	5.4 (1.8)
**Neuroimaging characteristics**	
Presence of lacunes	114 (15%)
White matter hyperintensities volume, ml^a^	1.0 (0.3, 2.9)
Total brain volume, ml	1070.6 (102.5)
Brain parenchymal fraction, %	76.3 (3.1)
**DTI and network connectivity parameters**	
Mean global fractional anisotropy	0.37 (0.02)
Mean global mean diffusivity, ×10^–3^ mm^2^/s	0.84 (0.05)
Network strength	0.06 (0.01)
Network density	0.11 (0.01)
Global efficiency	0.003 (0.001)

Data are mean (standard deviation) or frequency (percentage), unless otherwise specified.

*MMSE* mini-mental state examination, *DTI* diffusion tensor imaging.

^a^White matter hyperintensities volume is displayed as median (25th percentile, 75th percentile).

### White matter integrity and motor performance

**Figure 1:**
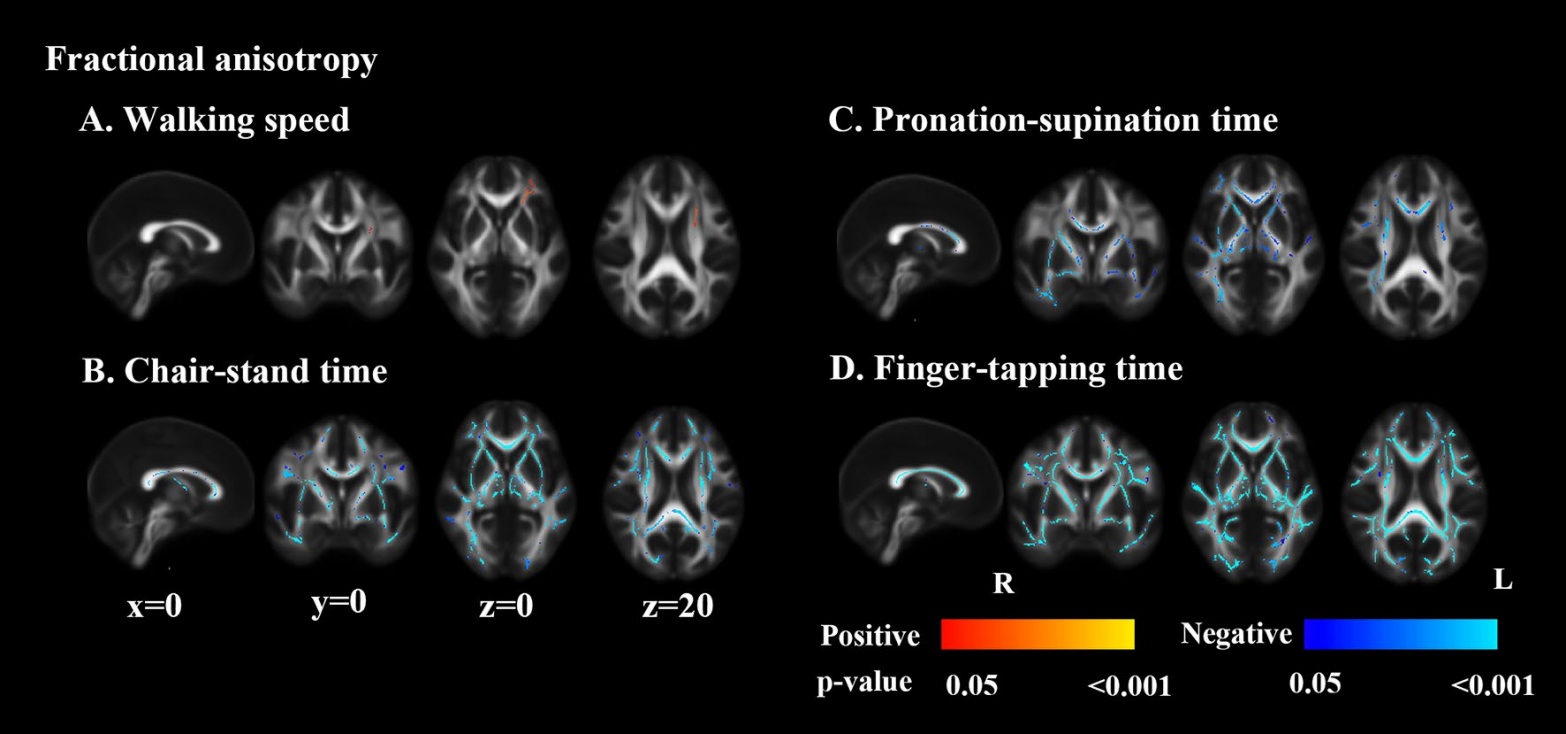
Tract-based spatial statistics of fractional anisotropy and motor performance. Decreased fractional anisotropy was associated with slower walking speed (**A**), longer 5-repeat chair-stand time (**B**), 10-repeat pronation-supination time (**C**), and 10-repeat finger-tapping time (**D**). Models adjusted for age, sex, height (in walking speed and chair-stand models), MMSE, presence of lacunes, white matter hyperintensities volume, and brain parenchymal fraction. All results were significant at p < 0.05 (threshold-free cluster enhancement corrected) and overlaid on mean FA map in Montreal Neurological Institute normalized space. The orange and blue lines indicate positive and negative associations between fractional anisotropy and motor parameters. X, y, and z indicate the coordinates.

As is shown in Fig. 1, lower FA in the left anterior thalamic radiation and left inferior fronto-occipital fasciculus was related to slower walking speed. Decreased FA in the corpus callosum, bilateral internal capsule, external capsule, corona radiate, and right posterior thalamic radiation related to longer time of 5-repeat hand pronation-supination. The lower FA at almost all voxels on the skeleton was associated with poor performance in chair-stand and finger-tapping tests. These associations were independent of the presence of lacunes, white matter hyperintensities, and global brain atrophy. Scatter plots of mean FA values across the significant TBSS skeleton versus the motor functions were shown in Supplementary Fig. 2. The MD maps show a similar pattern (Supplementary Fig. 3).

### Network connectivity and motor performance

As is shown in Table 2, greater network density, network strength, and global efficiency were associated with better performance in chair-stand, pronation-supination, and finger-tapping (less time-consuming) after adjustment for demographic and cognitive variables (model 1). Additional adjustment for the presence of lacunes, WMH volume, and brain parenchymal fraction (model 2) attenuated these associations, however, the associations of network connectivity with pronation-supination and finger-tapping were still significant (the model F-value and model p-value were shown in Supplementary Table 2). 10–13% variants of motor performance were explained by age, cognition, and cerebral structural injuries. The associations of network connectivity measures with upper-extremity motor performance (absolute standardized β from 0.121 to 0.155) were generally larger than with lower-extremity motor performance (absolute standard β from 0.034 to 0.085).

**Table 2:** Association of structural network connectivity with motor performance.

	Walking speed			5-repeat chair-stand time			10-repeat pronation-supination time			10-repeat finger-tapping time		
	Standard β	p	R^2^	Standard β	p	R^2^	Standard β	p	R^2^	Standard β	p	R^2^
**Model 1**												
Network density	0.094	0.016	0.110	− 0.114	0.004	0.090	− 0.179	< .001	0.122	− 0.132	0.002	0.113
Network strength	0.072	0.070	0.108	− 0.101	0.012	0.087	− 0.158	< .001	0.114	− 0.122	0.005	0.111
Global efficiency	0.076	0.054	0.108	− 0.099	0.013	0.087	− 0.155	< .001	0.114	− 0.131	0.002	0.112
**Model 2**												
Network density	0.060	0.161	0.111	− 0.085	0.050	0.096	− 0.155	< 0.001	0.136	− 0.132	0.002	0.118
Network strength	0.034	0.433	0.110	− 0.068	0.122	0.095	− 0.121	0.005	0.130	− 0.122	0.005	0.116
Global efficiency	0.041	0.331	0.110	− 0.070	0.099	0.095	− 0.125	0.003	0.131	− 0.131	0.002	0.119

standard β = standardized regression coefficient. R^2^ = model adjusted coefficient of determination.

Model 1: adjusted for age, sex, height (in walking speed and chair-stand models), and MMSE.

Model 2: adjusted for age, sex, height (in walking speed and chair-stand models), MMSE, presence of lacunes, white matter hyperintensities volume, and brain parenchymal fraction.

**Figure 2:**
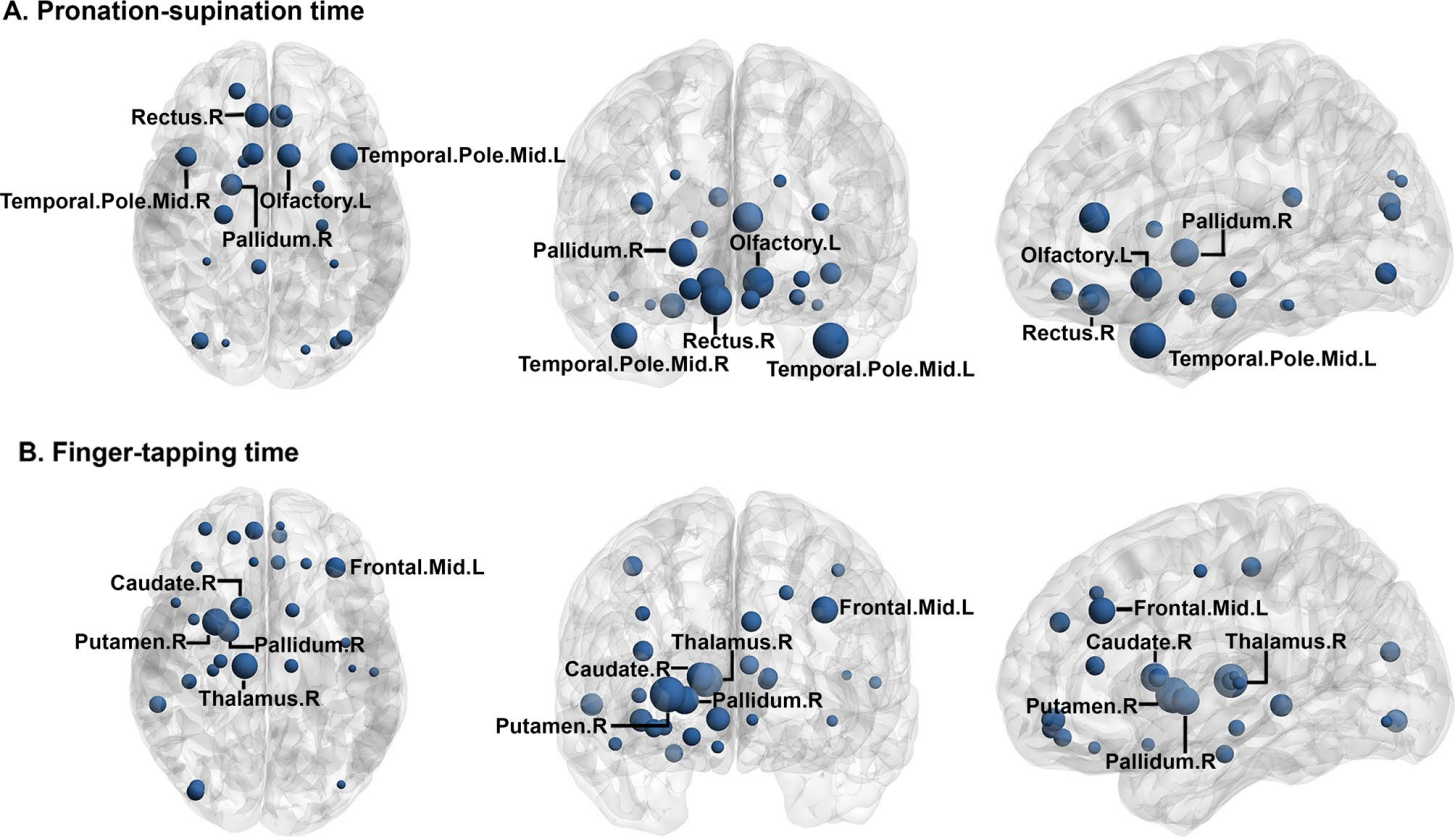
The association between nodal efficiency and upper-extremity motor performance. Nodes indicate cortical and subcortical brain regions according to Automated anatomical labeling atlas. The size of the nodes reflects the magnitude of the partial correlation coefficients. All nodes listed are significant after correction for multiple testing using Benjamini–Hochberg procedure at false discovery rate 0.05. We only labeled the top 5 most significant nodes. More details are shown in supplementary Table 2. The figure was drawn using BrainNet Viewer (https://www.nitrc.org/projects/bnv/).

In a post-hoc analysis, we calculated partial correlation coefficients between 90 nodal efficiency and each motor parameter. Figure 2 shows the spatial distribution of nodes that were significantly correlated to motor performance. Nodes (brain regions) with the strongest association with upper-extremity motor performance were primarily located in the basal ganglia, frontal, and temporal regions, with partial correlation coefficients ranging between − 0.089 and − 0.141 (Supplementary Table 3). Because we did not correct distortion on DTI, the findings should be interpreted with caution, especially for the nodes in the frontal and temporal pole. No node showed a significant association with walking speed and chair-stand time.

To validate the robustness of our results, we also analyzed left- and right-hand pronation-supination and finger-tapping, respectively, instead of the averaged score. The results were almost the same (Supplemental Fig. 4). Further adjustment for cardiovascular risk factors did not change the results (Supplemental Table 4 and Supplemental Fig. 5).

## Discussion

Our study shows that disrupted white matter integrity and reduced cerebral network connectivity were associated with motor performance. These associations were independent of lacunes, WMH, and global brain atrophy, suggesting that the impact of white matter injury on motor performance not only depends on these visible lesions on conventional MRI but also the microstructural disruption that is not captured on the currently used conventional MRI. Furthermore, compared with lower-extremity motor performance, there were generally stronger associations between network connectivity and upper-extremity motor performance.

Some previous studies have investigated the association of DTI parameters and lower extremity functions. The Whitehall II study^56^ (n = 387, mean age 69 years), a cohort of British civil servants, found a significant association of increased radial diffusivity and axial diffusivity in corona radiata and corpus callosum with poor chair-stand performance, but no relationship between DTI metrics and walking time. Our study found more widespread white matter involvement even additional adjustment for WMH, lacunes, and brain atrophy. We found decreased FA in the left anterior thalamic radiation and left inferior fronto-occipital fasciculus, primarily located in the frontal lobe, was related to slower walking speed. We also found decreased FA at almost all voxels on the skeleton was associated with poor chair-stand performance. This discrepancy across studies may partially be explained by participants’ characteristics and sample size. Compared with the Whitehall II study, our study was conducted in a rural community-dwelling sample with higher vascular risk burden (hypertension 50% vs. 30.2%; diabetes mellites 16% vs. 8.2%, current smoking 22% vs. 5.2%)^57^, so participants in our study probably bear more microstructural injury in the brain. Therefore, it may be the case that our study is sufficiently sensitive to detect the brain structure-motor relationship. Furthermore, the large sample size in our study may increase the power to find these associations. Besides, the RUN DMC study^18^ (n = 429, mean age 65.2 years), conducted in elderly patients with cerebral small vessel disease, found FA at almost all voxels on the skeleton were positively related to gait velocity; however, this relation attenuated after additional control for WMH and lacunar infarcts. The Rotterdam community-based study^58^ (n = 2330, mean age 65.9 years) found microstructure in thalamic radiation and association tracts were associated with worse gait peace (stride length and velocity) based on tract-based measurements. These findings were consistent with our results.

A few previous studies have investigated the association of white matter structure and upper extremity functions. A population-based study^23^ and a study conducted in families with a history of early-onset coronary artery disease^22^ found greater WMH volume was associated with decreased manual dexterity (Grooved Pegboard Test). However, the two studies did not assess white matter microstructural integrity. A small sample size study^24^ found white matter microstructure in the corticospinal tract and corpus callosum correlated with finger-tapping task learning scores. Another small study^25^ investigated the finger-specific descending motor system using probabilistic fiber tractography and found white matter integrity mediated the age-related motor performance. In our study, we found that decreased FA and increased MD in the corpus callosum, internal capsule, external capsule, and corona radiate related to poor performance in hand pronation-supination tasks. We also found widespread disrupted white matter integrity were associated with poor performance in finger tapping tasks. In the analysis of network connectivity, network density, network strength, and global efficiency were all significantly associated with upper extremity function, whereas not associated with walking and chair-stand performance. Our findings need to be confirmed and replicated in other samples.

In this study, we also found that more fiber tracts were correlated to chair-stand, hand pronation-supination, and finger-tapping tasks compared with usual speed walking. Global network connectivity parameters were associated with hand and finger tasks, but not associated with walking speed. These findings imply that the usual walking task might be insufficiently sensitive to detect fine deteriorations in mobility. More challenging tasks, such as repeated standing from a chair, pronating and supinating the hand, or tapping fingers as fast as possible, may offer more sensitive alternatives for assessment of early and subtle motor dysfunction. What’s more, in the analysis of nodal efficiency, we found that reduced nodal efficiency in basal ganglia, frontal, and temporal regions were associated with poor performance in hand pronation-supination and finger-tapping tasks. The findings should be interpreted with caution. Because we did not acquire reversed phase encoding images, we cannot correct distortion in subsequent processing steps. Therefore, we cannot exclude the possibility that the significant findings in frontal and temporal pole were false-positive results caused by brain distortion. Even though some unreliable regions at the brain edge, especially near the sinuses, some deep nodes which are less affected by distortion, such as pallidum, thalamus, and putamen, could be regarded to be truly significant findings. These deep nuclei have been proven to play important roles in motor control.

Motor control requires proper integration of various inputs and accurate communication between different brain regions, such as the motor cortex, somatosensory cortex, and basal ganglia^27,29^. Disrupted white matter integrity could dampen the communication efficiency and result in poor motor performance. We, therefore, could speculate that motor deficits in the aging population might be regarded as a “disconnection syndrome,” as proposed in cognitive decline^50,59–61^. Diffusion tensor imaging enables assessment of the microstructural organization of white matter, which may explain heterogeneous motor symptoms among individuals with a similar radiological degree of small vessel disease-associated features on MRI^31^.

The strengths of this study include the large sample size and the population-based setting, ensuring that our findings contribute to a general understanding of the involvement of white matter injury in motor control. Furthermore, we quantitatively evaluated both upper- and lower-extremity motor performance and employed a high-resolution MRI protocol to ensure the accuracy and validity of the study. Some limitations also need to be considered. First, the present analysis is based on a cross-sectional design, which does not allow for causal inferences. Longitudinal studies are needed to examine changes in white matter microstructure and motor decline. Second, participants with complete DTI and assessments of motor performance were generally younger, healthier, and had better cognition, which might have caused some selection bias. Third, even though probabilistic tractography is superior to deterministic tractography in many cases, given more computational demands of probabilistic tractography, we choose to apply deterministic tractography. We hope our findings would be validated in future studies. Fourth, because the deterministic fiber tracking technique is not valid for brainstem-crossing fibers and cerebellar connections, the cerebellum and brainstem were excluded in this study, although they were essential structures in motor control. Fifth, we used FA to represent white matter integrity but this is an over-simplification. FA is related to many factors, including crossing fibers, axonal density, degree of myelination, and fiber tract organization^62^. It is non-specific and cannot distinguish these factors. The exact neuro-mechanism of motor control warrants further studies. Sixth, we did not carry out distortion correction on DTI, so we cannot exclude the possibility that the significant findings in frontal and temporal pole were false-positive results caused by brain distortion. Seventh, although we excluded participants with significant motor signs, such as tremor and chorea, we cannot exclude the possibility that a few participants might have had early-stage degenerative disorders that may have caused minor Parkinson’s disease-like symptoms or other motor dysfunction. However, because of the low prevalence of these diseases in the general population, inclusion of a few of these patients would probably not change the present results.

## Conclusions

In conclusion, disrupted white matter integrity and reduced cerebral network connectivity were associated with poor motor performance. Diffusion-based methods provide a more in-depth insight into the neural basis of motor dysfunction.

## Supplementary information

Supplementary Information.
