# Supplementary material for: Disrupted white matter integrity and network connectivity are related to poor motor performance
Source: Sci Rep. 2020 Oct 27;10:18369. doi: 10.1038/s41598-020-75617-1 (PMC7591496; doi:10.1038/s41598-020-75617-1)
Supplement: Supplementary file 1 — Supplementary Information. [file 41598_2020_75617_MOESM1_ESM.docx]

**Disrupted white matter integrity and network connectivity are related to poor motor performance**

Feifei Zhai, MD^1^; Jie Liu, PhD^2,3^; Ning Su, MD^1^; Fei Han, MD^1^; Lixin Zhou, MD^1^; Jun Ni, MD, PhD^1^; Ming Yao, MD^1^; Shuyang Zhang, MD, PhD^4^; Zhengyu Jin, MD^5^; Liying Cui, MD, PhD^1^; Feng Tian, PhD^2,3^; Yicheng Zhu, MD, PhD^1*^

**SUPPLEMENTAL MATERIALS**

**Supplementary Tables**

Table 1. Correlations between motor performance, white matter integrity, network connectivity and covariates.

Table 2. Association of structural network connectivity with motor performance (model statistics).

Table 3. Correlation of nodal efficiency and upper-extremity motor performance.

Table 4. Association of structural network connectivity with motor performance, additionally adjusted for cardiovascular risk factors.

**Supplementary Figures**

Fig. 1. The average connectivity matrix.

Fig. 2. Scatter plots of the mean FA values across the significant TBSS skeleton versus the motor functions.

Fig. 3. Tract-based spatial statistics of mean diffusivity and motor performance.

Fig. 4. Association of fractional anisotropy with left- and right-hand pronation-supination and finger-tapping time.

Fig. 5. Association of fractional anisotropy and motor performance, additionally adjusted for cardiovascular risk factors.

**Table 1**. **Correlations between motor performance, white matter integrity, network connectivity and covariates.**

|  | Age | Sex | MMSE | Presence of lacunes | WMH volume | BPF |
| --- | --- | --- | --- | --- | --- | --- |
| Walking speed | -0.266  <0.0001 | 0.143  <0.0001 | 0.234  <0.0001 | -0.123  0.0007 | -0.163  <0.0001 | 0.220  <0.0001 |
| Chair-stand time | 0.261  <0.0001 | 0.018  0.614 | -0.221  <0.0001 | 0.136  0.0002 | 0.242  <0.0001 | -0.226  <0.0001 |
| Pronation-supination time | 0.200  <0.0001 | 0.023  0.532 | -0.294  <0.0001 | 0.091  0.012 | 0.253  <0.0001 | -0.212  <0.0001 |
| Finger-tapping time | 0.270  <0.0001 | 0.018  0.617 | -0.248  <0.0001 | 0.067  0.064 | 0.233  <0.0001 | -0.251  <0.0001 |
| Mean FA | -0.530  <0.0001 | -0.193  <0.0001 | 0.227  <0.0001 | -0.380  <0.0001 | -0.581  <0.0001 | 0.638  <0.0001 |
| Mean MD | 0.600  <0.0001 | 0.301  <0.0001 | -0.230  <0.0001 | 0.443  <0.0001 | 0.662  <0.0001 | -0.734  <0.0001 |
| Network density | -0.471  <0.0001 | 0.001  0.979 | 0.266  <0.0001 | -0.286  <0.0001 | -0.404  <0.0001 | 0.568  <0.0001 |
| Network strength | -0.455  <0.0001 | 0.137  0.0001 | 0.290  <0.0001 | -0.233  <0.0001 | -0.363  <0.0001 | 0.552  <0.0001 |
| Global efficiency | -0.419  <0.0001 | 0.189  <0.0001 | 0.279  <0.0001 | -0.214  <0.0001 | -0.299  <0.0001 | 0.486  <0.0001 |
| Age |  | 0.079  0.289 | -0.362  <0.0001 | 0.284  <0.0001 | 0.567  <0.0001 | -0.693  <0.0001 |
| Sex |  |  | 0.106  0.004 | 0.164  <0.0001 | 0.257  <0.0001 | -0.133  0.0002 |
| MMSE |  |  |  | -0.050  <0.0001 | -0.183  <0.0001 | 0.269  <0.0001 |
| Presence of lacunes |  |  |  |  | 0.396  <0.0001 | -0.364  <0.0001 |
| WMH volume |  |  |  |  |  | -0.601  <0.0001 |

Abbreviations: MMSE = mini-mental state examination, WMH = white matter, BPF = Brain parenchymal fraction, FA = fractional anisotropy, MD = mean diffusivity,

Data represent Pearson correlation coefficients for continuous-continuous variables, point biserial correlation coefficient for continuous-categorical variables (sex and presence of lacunes with other variables), and Cramer’s V for categorical-categorical variables (between sex and presence lacunes). The numbers in the second line are the corresponding p-values.

**Table 2. Association of structural network connectivity with motor performance (model statistics).**

|  | **Walking speed** | |  | **5-repeat chair-stand time** | |  | **10-repeat pronation-supination time** | |  | **10-repeat finger-tapping time** | |
| --- | --- | --- | --- | --- | --- | --- | --- | --- | --- | --- | --- |
|  | Model F | Model p |  | Model F | Model p |  | Model F | Model p |  | Model F | Model p |
| **Model 1** |  |  |  |  |  |  |  |  |  |  |  |
| **Network density** | 19.38 | <.001 |  | 15.63 | <.001 |  | 26.68 | <.001 |  | 24.60 | <.001 |
| **Network strength** | 18.88 | <.001 |  | 15.18 | <.001 |  | 24.82 | <.001 |  | 24.20 | <.001 |
| **Global efficiency** | 18.94 | <.001 |  | 15.14 | <.001 |  | 24.77 | <.001 |  | 24.46 | <.001 |
| **Model 2** |  |  |  |  |  |  |  |  |  |  |  |
| **Network density** | 12.60 | <.001 |  | 10.89 | <.001 |  | 17.71 | <.001 |  | 15.20 | <.001 |
| **Network strength** | 12.41 | <.001 |  | 10.69 | <.001 |  | 16.80 | <.001 |  | 14.94 | <.001 |
| **Global efficiency** | 12.45 | <.001 |  | 10.73 | <.001 |  | 17.00 | <.001 |  | 15.23 | <.001 |

Abbreviations: standard β = standardized regression coefficient. R^2^ = model adjusted coefficient of determination.

Model 1: adjusted for age, sex, height (in walking speed and chair-stand models), and MMSE.

Model 2: adjusted for age, sex, height (in walking speed and chair-stand models), MMSE, presence of lacunes, white matter hyperintensities volume, and brain parenchymal fraction.

**Table 3. Correlation of nodal efficiency and upper-extremity motor performance.**

| 10-repeat hand pronation-supination time | | |  | 10-repeat finger-tapping time | | |
| --- | --- | --- | --- | --- | --- | --- |
| Nodes | Partial correlation coefficient | Raw p |  | Nodes | Partial correlation coefficient | Raw p |
| Temporal.Pole.Mid.L | -0.141 | <0.001 |  | Putamen.R | -0.144 | <0.001 |
| Rectus.R | -0.131 | <0.001 |  | Thalamus.R | -0.140 | <0.001 |
| Olfactory.L | -0.130 | <0.001 |  | Pallidum.R | -0.129 | <0.001 |
| Pallidum.R | -0.128 | <0.001 |  | Caudate.R | -0.129 | <0.001 |
| Temporal.Pole.Mid.R | -0.124 | <0.001 |  | Frontal.Mid.L | -0.122 | <0.001 |
| Olfactory.R | -0.123 | <0.001 |  | Frontal.Med.Orb.R | -0.116 | 0.001 |
| Cingulum.Ant.L | -0.118 | 0.001 |  | Occipital.Inf.R | -0.115 | 0.002 |
| Frontal.Sup.Orb.R | -0.117 | 0.001 |  | Temporal.Mid.R | -0.110 | 0.003 |
| ParaHippocampal.R | -0.114 | 0.002 |  | Postcentral.R | -0.109 | 0.003 |
| Occipital.Inf.L | -0.114 | 0.002 |  | Frontal.Sup.Orb.R | -0.107 | 0.003 |
| Occipital.Mid.R | -0.113 | 0.002 |  | Occipital.Mid.R | -0.107 | 0.004 |
| Rectus.L | -0.109 | 0.003 |  | Frontal.Sup.Medial.L | -0.106 | 0.004 |
| Caudate.R | -0.105 | 0.004 |  | Frontal.Mid.Orb.R | -0.105 | 0.004 |
| Occipital.Mid.L | -0.104 | 0.004 |  | Caudate.L | -0.105 | 0.004 |
| Amygdala.L | -0.103 | 0.005 |  | Thalamus.L | -0.104 | 0.005 |
| Hippocampus.L | -0.101 | 0.006 |  | ParaHippocampal.R | -0.100 | 0.006 |
| Cingulum.Post.R | -0.098 | 0.008 |  | Cingulum.Ant.L | -0.096 | 0.009 |
| Occipital.Sup.L | -0.097 | 0.008 |  | Frontal.Sup.L | -0.096 | 0.009 |
| Fusiform.R | -0.095 | 0.010 |  | Hippocampus.R | -0.096 | 0.009 |
| Temporal.Pole.Sup.R | -0.094 | 0.011 |  | Insula.R | -0.095 | 0.009 |
| Fusiform.L | -0.092 | 0.012 |  | Temporal.Pole.Sup.R | -0.095 | 0.010 |
| Occipital.Sup.R | -0.092 | 0.012 |  | Frontal.Mid.R | -0.093 | 0.011 |
|  |  |  |  | Precentral.L | -0.093 | 0.011 |
|  |  |  |  | Temporal.Sup.L | -0.091 | 0.013 |
|  |  |  |  | Heschl.L | -0.091 | 0.013 |
|  |  |  |  | Rectus.R | -0.091 | 0.014 |
|  |  |  |  | Frontal.Med.Orb.L | -0.089 | 0.015 |
|  |  |  |  | Occipital.Inf.L | -0.089 | 0.015 |

Automated Anatomical Labeling template was employed to parcellate the cerebral cortex into 90 cortical and subcortical regions (45 for each hemisphere). All regions listed above are significant using the Benjamini-Hochberg procedure at a false discovery rate of 0.05.

**Table 4. Association of structural network connectivity with motor performance, additionally adjusted for cardiovascular risk factors.**

|  | **Walking speed** | |  | **5-repeat chair-stand time** | |  | **10-repeat pronation-supination time** | |  | **10-repeat finger-tapping time** | |
| --- | --- | --- | --- | --- | --- | --- | --- | --- | --- | --- | --- |
|  | Standard β | p |  | Standard β | p |  | Standard β | p |  | Standard β | p |
| Network density | 0.064 | 0.144 |  | **-0.094** | **0.034** |  | **-0.164** | **<.001** |  | **-0.131** | **0.003** |
| Network strength | 0.039 | 0.375 |  | -0.076 | 0.093 |  | **-0.132** | **0.003** |  | **-0.125** | **0.005** |
| Global efficiency | 0.047 | 0.270 |  | -0.078 | 0.073 |  | **-0.134** | **0.002** |  | **-0.131** | **0.002** |

Abbreviations: standard β = standardized regression coefficient.

Models are adjusted for age, sex, height (in walking speed and chair-stand models), MMSE score, white matter hyperintensities volume, presence of lacunes, brain parenchymal fraction, hypertension, diabetes mellites, hyperlipemia, and current smoking.


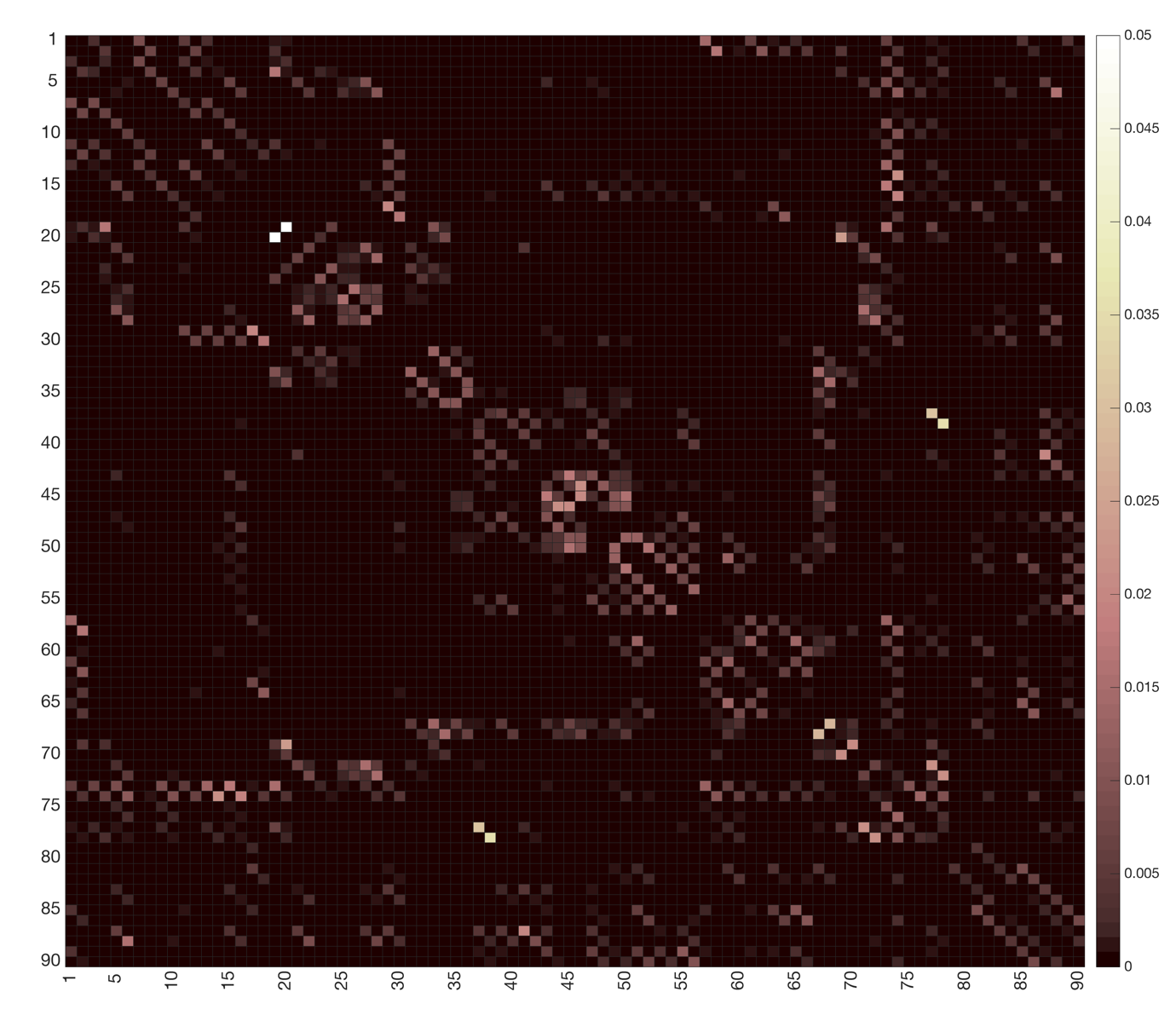


**Fig. 1. The average connectivity matrix.** Illustrative heatmaps of the average connectivity matrix of 90 brain regions according to automated anatomical labeling (AAL) atlas. Higher weighted edges between two regions are indicated by brighter color, with darker brown denoting weaker connectivity.

**
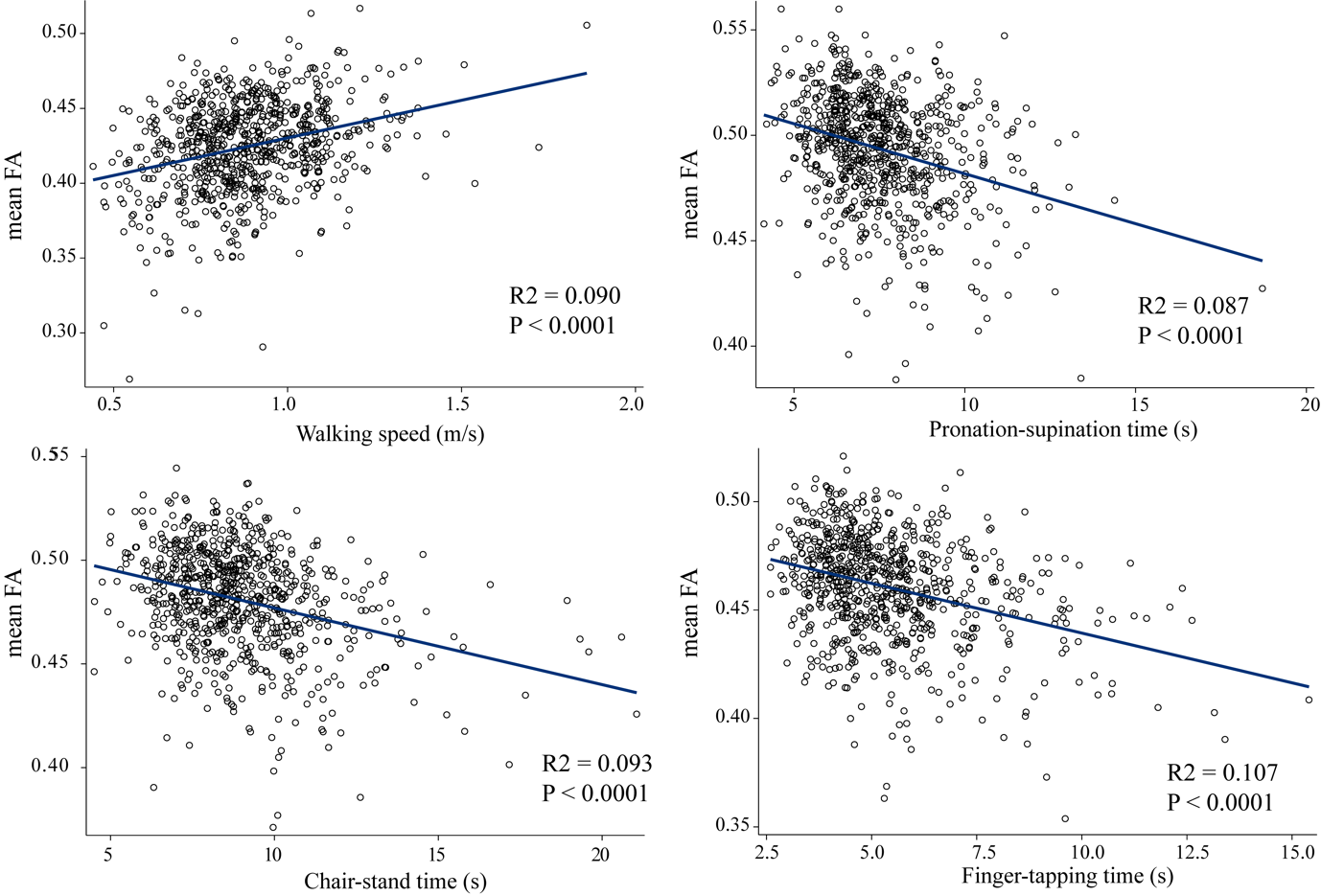
**

**Fig. 2. Scatter plots of the mean FA values across the significant TBSS skeleton versus the motor functions**. The best-fitting linear regression line is displayed on each scatter plot. R2 = coefficient of determination.

**
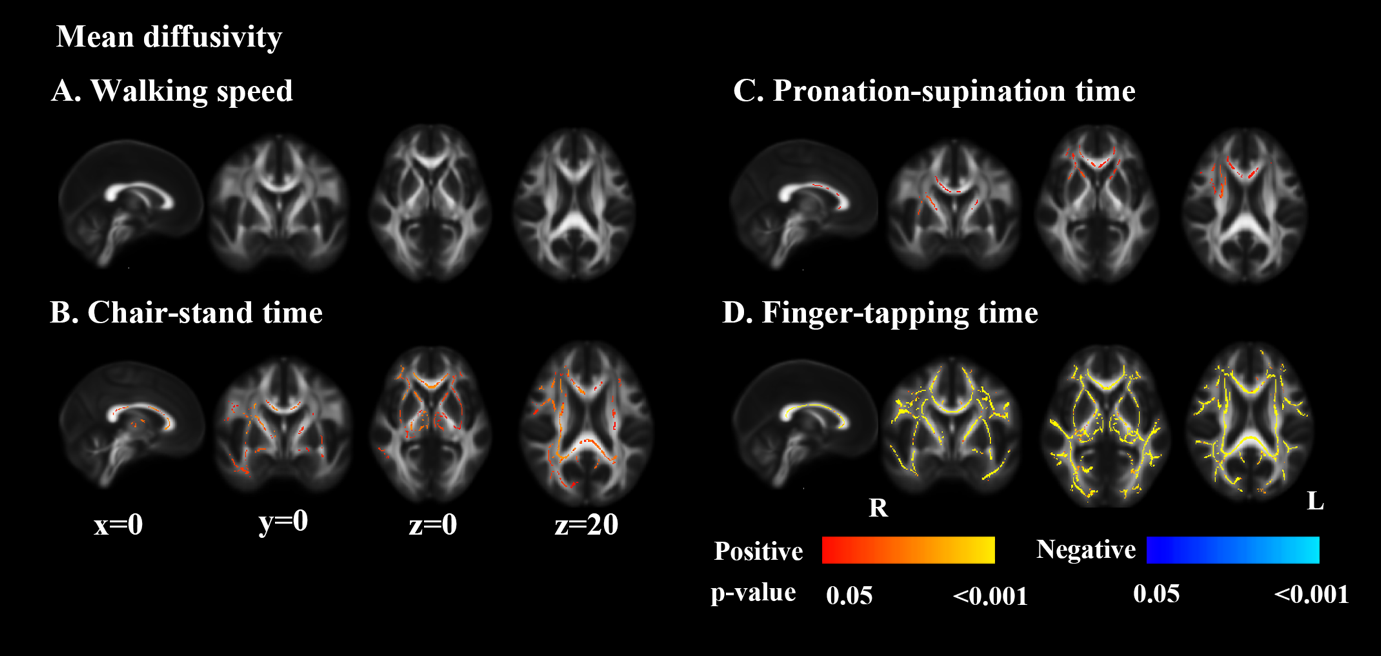
**

**Fig. 3. Tract-based spatial statistics of mean diffusivity and motor performance.**

Increased mean diffusivity was associated with longer 5-repeat chair-stand time (B), 10-repeat pronation-supination time (C), and 10-repeat finger-tapping time (D). Models adjusted for age, sex, height (in walking speed and sit-to-stand models), MMSE, presence of lacunes, white matter hyperintensities volume, and brain parenchymal fraction. All results were significant at p<0.05 (threshold-free cluster enhancement-corrected) and overlaid on the mean FA map in Montreal Neurological Institute normalized space. The red lines indicate positive associations between mean diffusivity and motor parameters. X, y, and z indicate the coordinates.


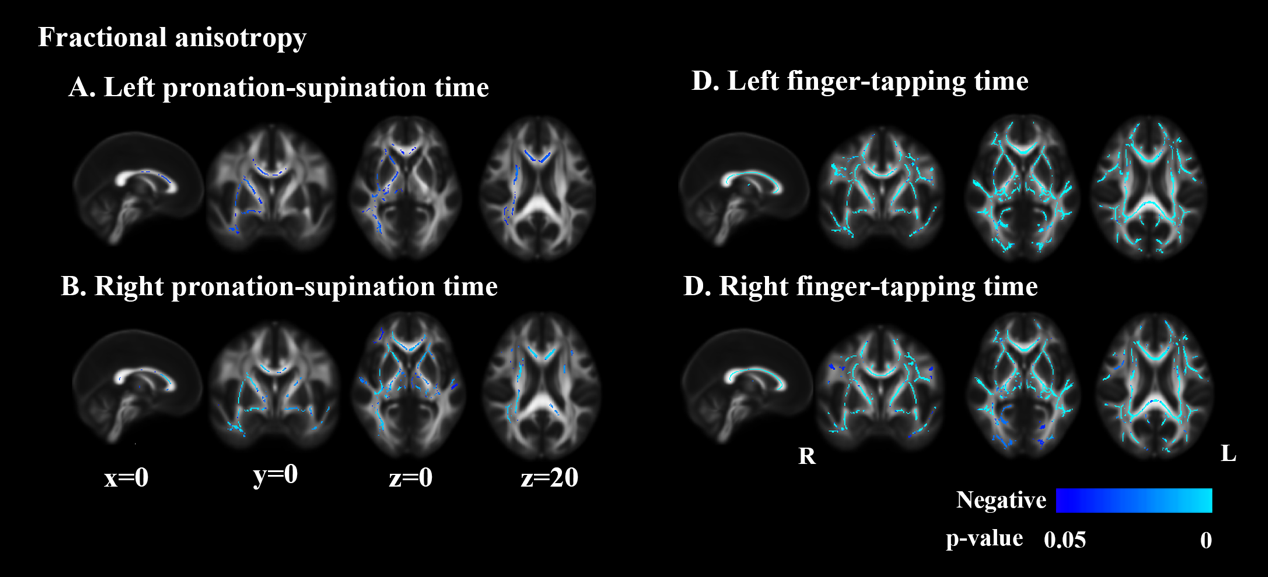


**Fig. 4. Association of fractional anisotropy with left- and right-hand pronation-supination and finger-tapping time.** Models adjusted for age, sex, MMSE, presence of lacunes, white matter hyperintensities volume, and brain parenchymal fraction. All results were significant at p<0.05 (threshold-free cluster enhancement-corrected) and overlaid on the mean FA map in Montreal Neurological Institute normalized space. The blue lines indicate negative associations between fractional anisotropy and motor parameters. X, y, and z indicate the coordinates.


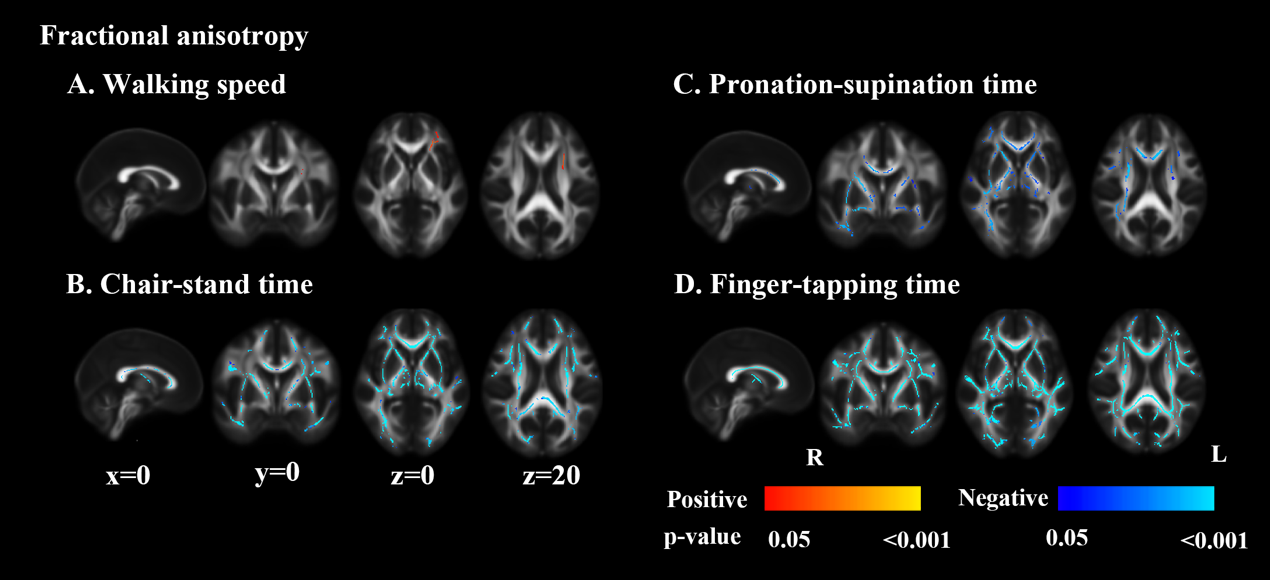


**Fig. 5. Association of fractional anisotropy and motor performance, additionally adjusted for cardiovascular risk factors.** Decreased FA was associated with slower walking speed (A), and longer 5-repeat chair-stand time (B), 10-repeat hand pronation-supination time (C), and 10-repeat finger-tapping time (D). Models adjusted for age, sex, height (in walking speed and sit-to-stand models), MMSE, white matter hyperintensities volume, presence of lacunes, brain parenchymal fraction, hypertension, diabetes mellites, hyperlipemia, and current smoking. All results were significant at p<0.05 (threshold-free cluster enhancement-corrected) and overlaid on the mean FA map in Montreal Neurological Institute normalized space. The red and blue lines indicate positive and negative associations between fractional anisotropy and motor parameters. X, y, and z indicate the coordinates.
